# Hepatitis B genotypes and surface antigen mutants present in Pakistani blood donors

**DOI:** 10.1371/journal.pone.0178988

**Published:** 2017-06-05

**Authors:** Barbara J. Harris, Vera Holzmayer, Huma Qureshi, Mohammad Ayyub Khan, Saleem Ahmed Khan, Nuzhat Salamat, Sarfaraz Jafri, Catherine A. Brennan

**Affiliations:** 1 Infectious Diseases Research, Abbott Diagnostics, Abbott Park, IL, United States of America; 2 Pakistan Medical Research Council, Islamabad, Pakistan; 3 Armed Forces Institute of Transfusion, Rawalpindi, Pakistan; 4 Army Medical College, Rawalpindi, Pakistan; 5 Hussaini Blood Bank, Karachi, Pakistan; University of Cincinnati College of Medicine, UNITED STATES

## Abstract

**Background:**

The prevalence of chronic Hepatitis B Virus (HBV) infection is 2–4% in the Pakistani population, defining Pakistan as an intermediate prevalence country. In this study, hepatitis B surface antigen (HBsAg) reactive blood donations were screened using a combination of serological and molecular methods to identify immune escape HBV mutant strains and to determine the HBV genotypes and subtypes present in Pakistan.

**Methods:**

Blood donations were collected at the Armed Forces Institute of Transfusion (AFIT) located in northern Pakistan and the Hussaini Blood Bank (HBB) located in the south. From 2009 to 2013 a total of 706,575 donations were screened with 2.04% (14,409) HBsAg reactive. A total of 2055 HBsAg reactive specimens, were collected and screened using a monoclonal antibody based research assay to identify immune escape mutants followed by PCR amplification and DNA sequencing to identify the mutation present. DNA sequences obtained from 192 specimens, including mutant candidates and wild type strains, were analyzed for escape mutations, genotype, and HBsAg subtype.

**Results:**

Mutations were identified in approximately 14% of HBsAg reactive donations. Mutations at HBsAg amino acid positions 143–145 are the most common (46%) with the mutation serine 143 to leucine the most frequently occurring change (28%). While regional differences were observed, the most prevalent HBV strains are subgenotypes of D with subgenotype D1/subtype ayw2 accounting for the majority of infections; 90.2% at AFIT and 52.5% at HBB.

**Conclusions:**

The high frequency of immune escape HBV mutants in HBV infected Pakistani blood donors highlights the need for more studies into the prevalence of escape mutants. Differences between vaccinated and unvaccinated populations, the correlation of escape mutant frequency with genotype, and impact of escape mutations in different genotype backgrounds on the performance of commercially available HBsAg assays represent avenues for further investigation.

## Introduction

Hepatitis B virus (HBV) is a global public health issue with 15–25% of chronically infected individuals developing severe disease and dying prematurely due to cirrhosis, hepatocellular carcinoma or hepatitis. The World Health Organization (WHO) estimates approximately 780,000 deaths per year among the 240 million people with chronic HBV infection [1]. Pakistan is classified as an intermediate prevalence country with a prevalence of 2–4% chronic HBV infection and an additional 32% of the population having had exposure to HBV [2]. The prevalence of chronic HBV infection varies between and within provinces, in rural versus urban populations, and by socioeconomic status. In a national survey conducted from 2007–2008, overall HBV surface antigen (HBsAg) prevalence was 2.5% with provincial rates ranging from 4.3% in the Balochistan province to 1.3% in the northern Khyber Pakhtunkhwa province with the southern province of Sindh and Punjab at 2.5% and 2.4% respectively [3]. Ali et al. [4] report prevalences ranging from 4 to 11% in regions of the Balochistan province compared to 1.5 to 7% in the Punjab province. A comparison of rural and urban populations showed HBV prevalence was 3.8 fold higher in the rural areas [5], while low socioeconomic status was associated with a 2.2 fold higher prevalence [5–7]. The major risk factors for acquisition of HBV in Pakistan are unsafe practices used for therapeutic injections and infusions and for barbering [2, 3, 7, 8]; males have a prevalence of HBV infection 2.2 fold higher than females, which is likely due to higher rates of injections and use of barbers [9].

Transfusion transmitted hepatitis is also a significant risk in Pakistan; the approximately 1.5 million blood products transfused annually are primarily replacement and directed donations with approximately 10%–15% collected from paid donors [4, 10, 11]. In addition, blood screening procedures are neither regulated nor standardized [4, 11]. However, it is notable that there has been a decline in HBsAg reactive donations [12, 13]. At the Armed Forces Institute of Transfusion (AFIT) 5.2% of donations were HBsAg reactive in 1996 declining to 1.27% in 2013 [12, 13]. The downward trend may be attributed to increased awareness, resulting in safer injection practices and use of disposable syringes, and to the screening of military recruits.

HBV is an enveloped virus with a partially double stranded circular DNA genome. HBV mutates rapidly due to replication via an error prone reverse transcriptase resulting in a high mutation rate (approximately 1 point mutation per round of replication); this error rate coupled with the high replication rate in chronically infected persons (>10^11^ virion per day) leads to evolution of HBV mutant strains [14]. Of particular concern are immune escape mutations that result in chronic HBV infection; escape mutants arise in response to vaccination, hepatitis immunoglobulin treatment, and naturally occurring neutralizing antibodies.

HBsAg forms the envelope of the virion, and antibodies against HBsAg provide immune protection either after vaccination or after acute infection and natural clearance of the virus. The major target for neutralizing antibodies is the “a” determinant comprising amino acids 99–160 of HBsAg [15]. Within the “a” determinant, two loops are formed by cysteines 107 and 138 (loop 1) and 139 and 147 (loop 2) with loop 2 being the primary target for vaccine induced antibodies [15]. Vaccine escape mutations have been identified from amino acid 116 through 145 [16]. Data is limited on the frequency of naturally occurring immune escape mutations.

HBsAg is the primary serological marker for diagnosis of acute and chronic HBV infection. Mutations in HBsAg can have an impact on performance of the commercially available assays used to detect HBsAg; reported mutations are primarily located at positions 120–124, 130–133, 142 and 144–145 [15, 17]. While current HBsAg tests have improved detection of “a” determinant mutants, there is still a need to monitor assay sensitivity for HBsAg mutants circulating in different populations and the impact of the genotype backbone on mutant detection.

The error prone replication of HBV has also lead to the evolution of 9 HBV genotypes. Currently, HBV is classified into genotypes A-H and a proposed genotype I with subgenotypes for A-D and F [18]. Genotypes differ from each other at the nucleotide level by >7.5% across the complete genome while subgenotypes differ >4% within a genotype. Genotypes evolved in different geographic regions but over time the distribution has diversified [18]. Genotypes A-D are the most prevalent strains and can be found throughout the world. However, genotype A prevalence is highest in Southern and Eastern Africa, genotype B is highest in Southeast and East Asia, genotype C is highest in East Asia and the Pacific Islands, and genotype D is highest in Northern Africa and West, Central, and South Asia. Genotypes E-H are less prevalent with genotype E found primarily in Central and West Africa, genotype F in Central and South America, and genotypes G and H in Central America.

Reports of the HBV genotypes circulating within Pakistan show conflicting results. A summary of 10 studies indicated that overall genotype D (63.7%) predominates in Pakistan but prevalence varied from 8% in one study to 100% in another [19]. The difference in genotype prevalence between these reports could be due to population differences or to the methodology used to determine genotype. Two studies that used HBV genotype-specific PCR reported very different results. In one, a molecular epidemiology study of patients from hospitals in 4 provinces reported predominantly genotype D (65.3%) followed by genotype B (26.7%) and genotype A (4.9%) [9]. In contrast, the other study reported predominantly genotype C (28%) followed by genotype B (18%), genotype A (14%), genotype D (13%), genotype E (0.6%) and genotype F (1.3%) [20]. For this latter study, the HBV strains were obtained primarily in the Punjab province where genotype C was the dominate strain; genotype C was also most prevalent in Khyber Paktoonkhwa while genotype B predominated in Balochistan and genotype A was most common in Sindh [20]. A third study that screened patients nationwide using a commercial probe-based genotyping assay found a genotype D prevalence of 96% [21].

In this study, HBsAg reactive blood donations were used to characterize HBV strains circulating in Pakistan. The donations were collected at two blood banks, one located in Rawalpindi, Punjab (North) and one in Karachi, Sindh (South). We performed a serological assay to identify HBsAg mutants and HBV PCR amplification followed by sequencing to characterize HBV strains found in infected blood donors. HBsAg sequences were evaluated to identify escape mutations and to determine genotype and subtype.

## Results

### Blood donor population

For this study, HBsAg reactive specimens, identified by routine blood-screening testing, were collected at the Armed Forces Institute of Transfusion (AFIT) and Hussaini Blood Bank (HBB) from 2009–2013. AFIT, a regional blood center located in northern Pakistan in the Punjab province, serves the armed forces hospitals in Rawalpindi and Islamabad, and civilian hospitals in the region [13]. A total of 267,918 blood donations were screened during the study of which 1.45% were reactive for HBsAg. The AFIT donor population was 99% male with a mean age of 28 (range 18–69 years). HBB is based in Karachi in the southern region of the Sindh province with locations in the cities of Karachi, Badin, and Jacobabad, and serves hospitals nationwide. A total of 438,657 blood donations were screened during the study of which 2.4% were reactive for HBsAg. The HBB donor population were 96% male with a mean age of 27 (range 18–61 years). A total of 2055 randomly selected reactive specimens with available volume were collected for this study; 957 from AFIT and 1098 from HBB.

### Screen for HBsAg mutants and molecular confirmation

**Fig 1:**
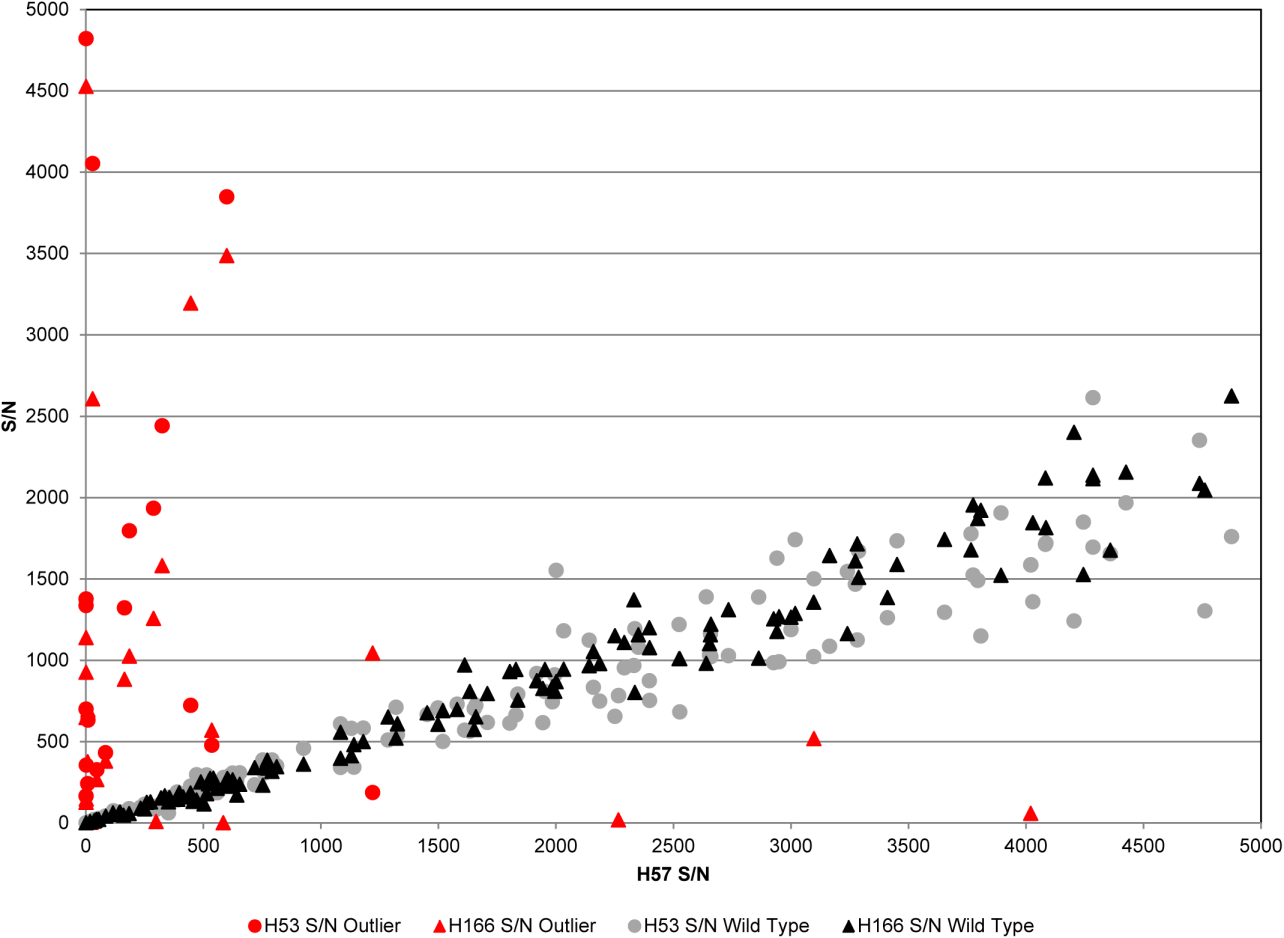
Representative results for the HBsAg mutant assay. S/N values for each specimen are plotted using MAb H53 and H166 values on the y-axis and MAb H57 values on the x-axis. MAb H53 values are shown by filled circles and MAb H166 are filled triangles; red indicates outlier data points identifying mutant candidate specimens, and gray and black indicate wild type data points.

To identify HBV strains harboring mutations in the surface antigen protein, donor specimens reactive for HBsAg were screened using a research assay designed to identify diagnostic and vaccine escape mutations based on differential binding to 3 mouse monoclonal antibodies (MAbs). The MAb H166 binds an epitope (E1) defined as the amino acids (aa) 121–124 within the “a” determinant loop 1 (aa 107–138) of HBsAg [22]. The MAb H57 binds an epitope (E2) within loop 2 (aa 139–147). The MAb H53 binds a conformational epitope that maps to discontinuous segments of HBsAg, aa 112–117 in the “a” determinant and aa 182–207 outside of the “a” determinant [22]. As illustrated in Fig 1, specimens that are nonreactive or have low reactivity to H166 but are reactive for H53 and H57 indicate a mutation in E1. Conversely, specimens that are nonreactive or have low reactivity to H57 but are reactive for H53 and H166 indicate a mutation in E2. Specimens nonreactive for all 3 MAbs may indicate the presence of mutations that cause major conformational changes to HBsAg or may be due to the HBsAg level being below the sensitivity of the assay. Specimens that are reactive to all 3 MAbs are classified as wild type. In Fig 1, the wild type specimens form a trend where the signal to noise (S/N) values for each MAb relative to the others forms a linear correlation. Specimens with H166 S/N values that fall below the wild type trendline close to the x-axis are classified as E1 mutants; i.e. H166 has very low or nonreactive value relative to H53 and H57 S/N values. Possible E1 mutants lay in between the x-axis and the trend line; i.e. H166 S/N value is low relative to H53 and H57 but is not nonreactive. Specimens classified as E2 mutants fall above the trendline close to the y-axis; i.e. S/N for H57 is very low or nonreactive while S/N values for H53 and H166 are relatively high. Possible E2 mutants lay between the y-axis and the trendline.

The HBsAg mutant assay was used to screen a total of 2055 donor specimens with 60 (2.9%) classified as mutants and possible mutants (Table 1). Seventy three (3.6%) specimens were nonreactive in the mutant assay and 1922 (93.5%) were classified as wild type. To confirm the presence of HBsAg mutations and identify the amino acid changes, PCR amplification followed by DNA sequencing was performed on the 60 specimens classified as mutants/possible mutants. Sequence for the “a” determinant of HBsAg was obtained from 55 of 60 (92%) mutant candidates (Table 1). HBsAg immune escape mutations were identified using the criteria of the Geno2pheno (hbv) v2.0 software [23]. Mutations at amino acid position 123 were not classified as escape mutations by Geno2pheno but were included in our analysis as they fall within E1. Some mutations that Geno2pheno classifies as escape mutations are actually common in a specific HBV genotype and were omitted from the count of mutations. Those omitted included R122K which is the determinant for subtyping HBsAg as ad versus ay, T126I for genotype C, A128V for genotype D2, and T131N for genotype A1.

**Table 1:** HBsAg mutant assay and PCR results.

HBsAg Mutant Assay Result	N =	Selected for PCR	PCR Positive	Wild Type Strains	Mutant Strains	# of Mutations in E1*	# of Mutations in E2	# of Mutations outside E1&E2^
E1 mutant	8	8	8**	3	4	4	1	0
E2 mutant	23	23	21	7	14	1	14	2
E1 + E2 mutant	1	1	1	0	1	1	0	0
possible E1 mutant	8	8	8	6	2	0	0	2
possible E2 mutant	20	20	17**	11	5	0	5	2
Nonreactive	73	62	38	30	8	2	4	5
Wild Type	1922	108	99	85	14	1	4	13
Total	2055	230	192	142	48	9	28	24

** A&D dual infection sequence was not analyzed

*includes P120X and T123S which are not escape mutations but are located within E1 epitope

^ omit T126I for genotype C, A128V for genotype D2, and T131N for genotype A1

For the E1 mutant candidates, 57% (4 of 7) confirmed to have mutations in the E1 epitope with one also having a mutation in the E2 epitope. One candidate was a dual heterogeneous infection of genotypes A and D; the mixed sequence prevented mutation analysis. E2 mutations were present in 66.7% (14 of 21) of E2 candidates with one also having a mutation in E1 and one having 2 mutations outside of E1 and E2. The specimen classified as both an E1 and E2 mutant had a single escape mutation in E1. Overall, for those PCR positive, mono-infected specimens the HBsAg mutant assay classified as mutants, 65.5% (19 of 29) were confirmed by DNA sequencing.

There were 8 specimens classified as possible E1 mutants and only 2 had escape mutations with both located outside of E1 and E2. Of the 20 possible E2 mutants, 1 was a dual heterogeneous genotype A and D infection and was not analyzed, 3 were PCR negative, and only 5 of the remaining 16 had escape mutations within E2 with one having 2 additional mutations outside of E1 and E2. Of 24 possible mutants, sequencing confirmed only 29.2% (7 of 24) as having HBsAg escape mutations.

For specimens that were nonreactive in the mutant assay, only 38 of 62 (61%, 11 of 73 had insufficient volume for PCR) were PCR positive and of these 21% (8 of 38) had escape mutations; 1 had 2 mutations within E1, 2 had mutations within E2, 1 had 2 escape mutations within E2 plus 1 outside of E1 and E2, and 4 had mutations outside of E1 and E2 (Table 1). The lower PCR positivity for nonreactive specimens is likely, in part, a reflection of assay sensitivity for HBsAg. For instance, many of these specimens had low levels of antigen and virus and thus were nonreactive in the mutant assay yet reactive for HBsAg in the more sensitive ARCHITECT HBsAg Qualitative assay used by the blood banks for blood screening.

To evaluate HBV mutations in Pakistani blood donors in an unbiased way, 108 specimens classified as wild type by the HBsAg assay and split roughly evenly between the two sites were randomly selected for PCR amplification and sequencing; 99 of 108 (92%) were PCR positive (Table 1). Surprisingly, 13.1% (14 of 99) of specimens harbored HBsAg escape mutations with 9 of the 14 (64%) having mutations outside of E1 and E2, 4 having mutations within E2 and 1 having a mutation within E1.

To estimate overall prevalence of HBV escape mutants in the Pakistan donor populations in this study, the bias of selection introduced by our mutant screening assay must be taken into account. Therefore the overall prevalence was extrapolated based on the percentage of mutants confirmed by DNA sequence in each mutant assay classification (i.e. mutant, possible mutant, nonreactive, and wild type) weighted by the percentage of the total population represented by each classification. From this calculation, we estimate 14% of HBsAg reactive donations harbor escape mutations in the study population.

**Fig 2:**
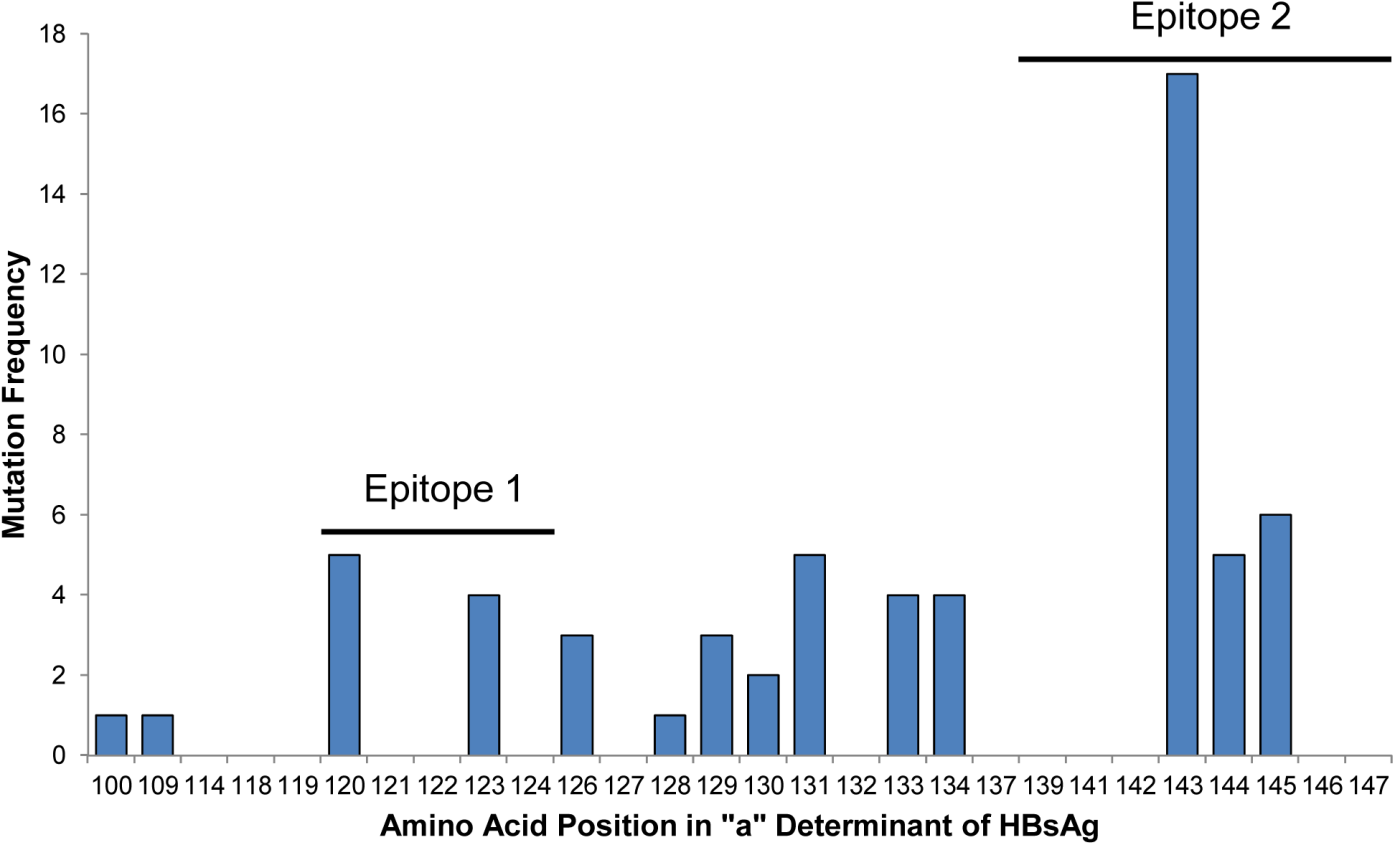
Histogram of mutation frequency. Shown is the frequency of mutations at each amino acid position within the HBsAg “a” determinant defined as sites of escape mutations by Geno2pheno v2.

Based on the HBV DNA sequences from 192 individuals, a total of 48 carried HBV strains containing escape mutations. Those 48 sequences contained a total of 61 escape mutations. The frequency of escape mutations at the 28 amino acid positions within the HBsAg “a” determinant that Geno2pheno classifies as escape mutations is shown in Fig 2; mutations were located at 14 amino acid positions within the “a” determinant of HBsAg. Mutations were most frequent within E2 with a total of 28 (46%) at positions 143–145 and the most frequently mutated position was serine 143 (n = 17; 28%). A total of 9 mutations were present within E1 while 24 mutations occurred outside of E1 and E2 (Fig 2).

The nomenclature to specify mutants used here is the one letter code for the wild type amino acid, the amino acid position within HBsAg and the one letter code for the mutant amino acid; for example, P120S designates proline at position 120 changed to serine. When the DNA sequence indicated more than one base call at a position, all possible amino acids were listed, such as P120PS for a mixture of proline and serine at position 120. Six strains had mutations exclusively in E1 including changes at P120, which were included with the E1 mutations because the 3 P120S mutants were all classified as E1 mutants in the HBsAg mutant assay. E1 mutants included P120S (n = 3), P120PS (n = 1), P120PT plus T123TI (n = 1) and T123S (n = 1). There were 2 strains that had mutations in both E1 and E2; T123TS plus S143L and T123TI plus S143SL. A total of 22 strains had mutations only within E2; S143L (n = 11), S143SL (n = 3), D144A (n = 1), D144E (n = 2), D144DG (n = 1), G145A (n = 2), and G145GR (n = 2). Three strains had mutations within E2 plus additional mutations outside of E1 and E2; S143SL, T131TN, and M133MT (n = 1), D144A, G145A, and M133I (n = 1), and G145R, T126TI, and Y134YN (n = 1). Finally 15 strains had mutations outside of E1 and E2; Y100C plus Q129R (n = 1), L109LR (n = 1), T126S (n = 1), T126TS (n = 1), A128V (n = 1), Q129H (n = 1), Q129QH (n = 1), G130R (n = 1), G130R, T131N and M133T (n = 1), T131N (n = 1), T131TN (n = 1), T131I (n = 1), T133TLMS (n = 1), Y134S (n = 1), and Y134YN (n = 1). A summary of the amino acid changes is shown in Table 2.

**Table 2:** Summary of HBsAg escape mutations.

Amino Acid Position	Native Amino Acid	Escape Mutation	Frequency
100	Tyr	Cys	1
109	Leu	Leu/Arg	1
120	Pro	Ser	3
		Pro/Ser	1
		Pro/Thr	1
123	Thr	Ser	1
		Thr/Ser	1
		Thr/Ile	2
126	Thr	Ser	1
		Thr/Ser	1
		Thr/Ile	1
128	Ala	Val	1
129	Gln	His	1
		Arg	1
		Gln/His	1
130	Gly	Arg	2
131	Thr	Asn	2
		Ile	1
		Asn/Thr	2
133	Met	Ile	1
		Thr	1
		Met/Thr	1
		Met/Leu/Ser/Thr	1
134	Tyr	Ser	1
		Asn/Tyr	2
		Tyr/Ala/Asp/Phe/Ser/Val	1
143	Ser	Leu	12
		Leu/Ser	5
144	Asp	Ala	2
		Glu	2
		Asp/Gly	1
145	Gly	Ala	3
		Arg	1
		Gly/Arg	2

For 37 of 61 (60.7%) mutations, there was a complete change to the mutant amino acid, while the remaining 24 (39.3%) had a mixture of wild type and mutant amino acids. Of note, for confirmed mutants classified as either mutant or possible mutant by the HBsAg mutant assay, 77% (20 of 26) of mutations in E1 and E2 were not mixtures of wild type and mutant amino acids. In contrast, only 5 of 14 (35.7%) escape mutants classified as wild type by the mutant assay had mutations in the E1 or E2 epitopes and 3 of the 5 had mixtures of wild type and mutant amino acids. Furthermore, 64% (9 of 14) of escape mutants with wild type classification by the HBsAg mutant assay had mutations outside of the E1 and E2 epitopes, with 4 having mixtures of wild type and mutant amino acids.

### Phylogenetic classification of HBV strains in donor populations

**Fig 3:**
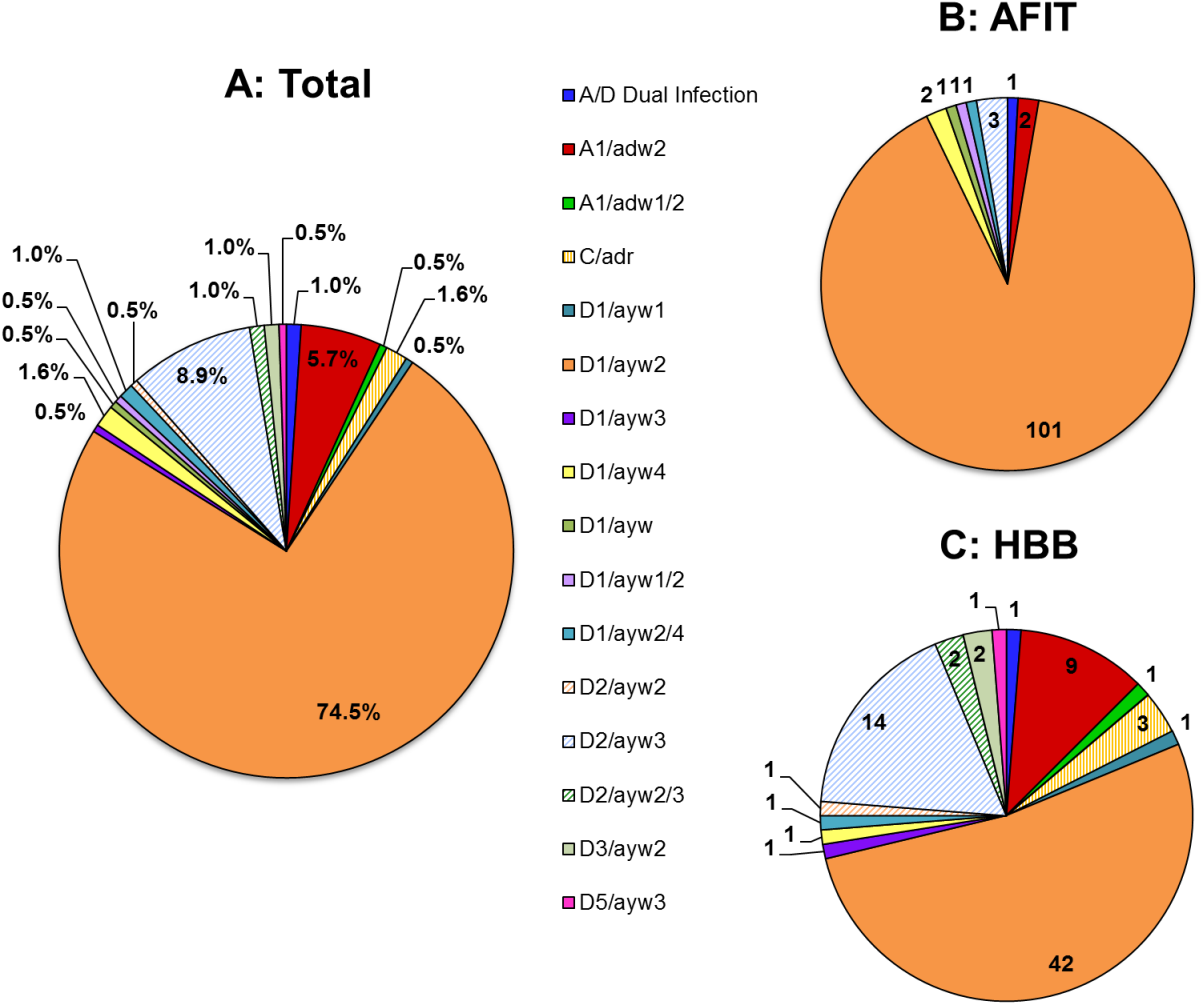
Prevalence of HBV genotype/subtype in donor population. (A) shows the percent of each genotype/subtype obtained from 192 HBV DNA sequences; 74.5% (143 of 192) were genotype D1/subtype ayw2. (B) shows the number of each genotype/subtype identified in the AFIT donor population; 101 of 112 (90.2%) were genotype D1/subtype ayw2 and 7 other genotype/subtype strains were present at low levels. (C) shows the number of each genotype/subtype identified in the HBB donor population; 42 of 80 (52.5%) were genotype D1/subtype ayw2, 14 (17.5%) were D2/ayw3, 9 (11.3%) were A1/adw2 and 10 other genotype/subtype strains were present at low levels. In all charts, the order of data labels in the legend corresponds to the clockwise order of the pie slices starting from the top.

**Fig 4:**
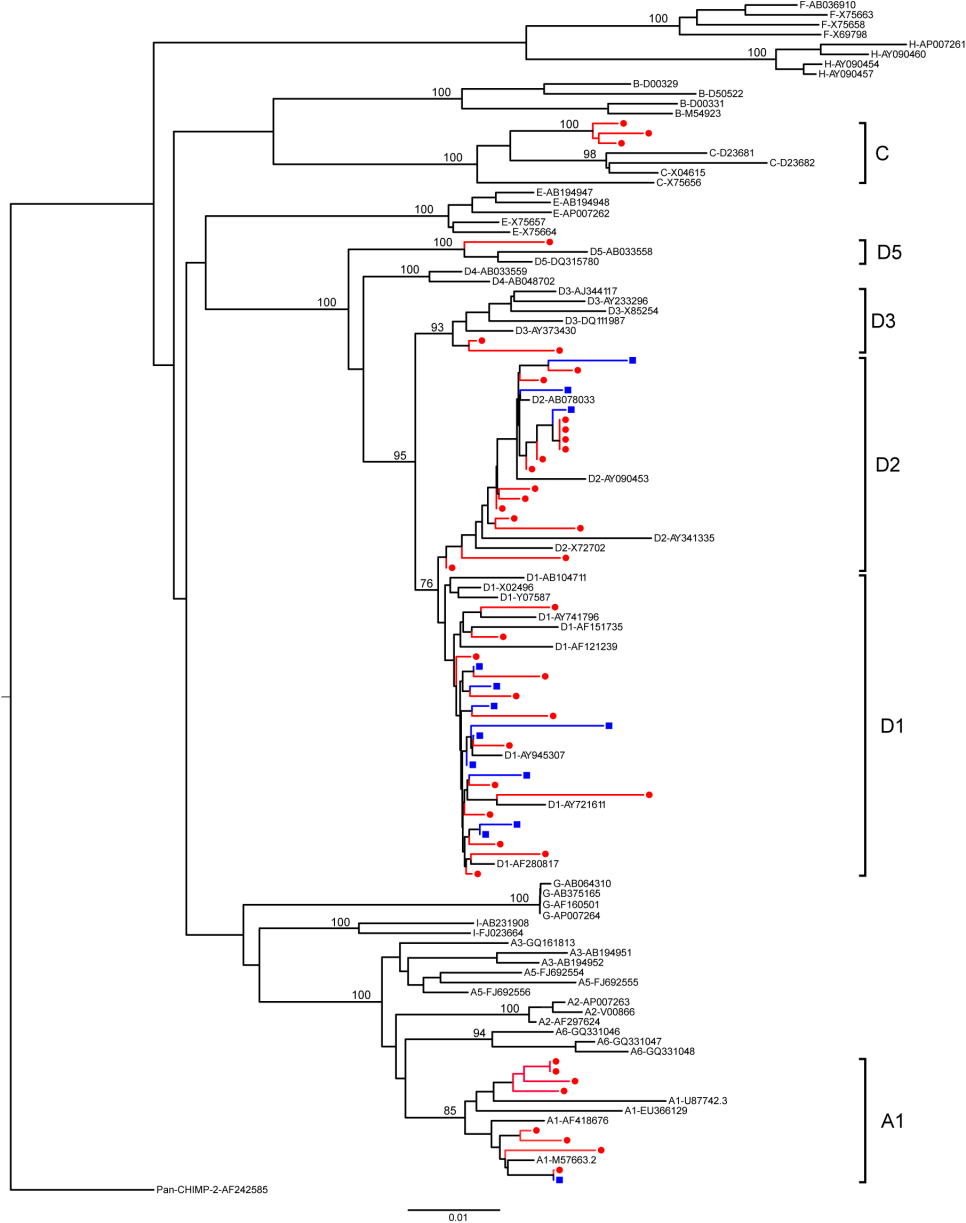
Phylogenetic tree of representative HBV strains. The phylogenetic tree was derived from an alignment of a subset of the HBV DNA sequences (n = 55) chosen to represent all sampling sites, collection years and genotypes identified in the donor population aligned with reference HBV sequences as described in Materials and Methods. Bootstrap values are shown for the major branches. Blue squares indicate sequences from AFIT donors and red circles indicate sequences from HBB donors. For improved visualization, the number of sequences was limited to a subset of the total.

The HBV DNA sequences obtained from 192 donor specimens were used to determine the genotypes and HBsAg subtypes circulating in the study population (Genbank accession numbers KT201157—KT201346, KY779733, KY779734). Genotypes present included A1, C, D1, D2, D3, and D5 and subtypes included adw, adr, and ayw. Overall, genotype D1/subtype ayw2 accounted for 74.5% of HBV infections, D2/ayw3 for 8.9%, A1/adw2 for 5.7% and 11 other genotype/subtype combinations were present at low levels (Fig 3A). While these top three categories accounted for the majority of strains at both sites, the strain diversity was quite different between the two blood banks. At AFIT, the vast majority of infections were due to D1/ayw2 which accounted for 90.2% (101 of 112) of strains present in the donor population; D2/ayw3 and A1/adw2 accounted for only 2.7% (n = 3) and 1.8% (n = 2) of infections, respectively (Fig 3B). In contrast, a greater diversity of strains was present at HBB; 52.5% (42 of 80) were D1/ayw2, 17.5% (14 of 80) were D2/Ayw3, and 11.3% (9 of 80) were A1/adw2 (Fig 3C). Genotypes C (n = 3), D3 (n = 2), and D5 (n = 1) were found at low frequencies and only at the HBB site. The phylogenetic tree shown in Fig 4, was constructed from a representative subset of sequences for the genotypes present in Pakistani donors to illustrate the extent of HBV genetic diversity circulating in the population. Of note, for genotypes present in both the AFIT and HBB donor populations, the tree shows no significant phylogenetic separation of geographically distinct sequences.

## Discussion

Screening of healthy blood donors at the AFIT and HBB blood banks in Pakistan showed a prevalence of 2.0% chronic HBV infection; prevalence was 1.4% at AFIT and 2.4% at HBB. Because the design of the study focused on chronic infections from HBsAg reactive donors, and would miss occult HBV infections, the true prevalence of HBV infection in Pakistan may be higher than observed in this study. While donor populations are typically considered representative of the general adult population, this is not true for our study as males accounted for 97% of the donors (99% at AFIT and 96% at HBB). Molecular characterization of a subset of the HBsAg reactive donor specimens revealed that approximately 14% of the HBV strains harbored immune escape mutations. This prevalence, among HBsAg reactive donors, may also be an under-representation of the true prevalence, as occult infections may result from mutations in the HBsAg “a” determinant. Phylogenetic analysis of the DNA sequences showed that genotype D predominated with subgenotype D1/subtype ayw2 as the most prevalent strain circulating in the study population.

We employed an HBsAg mutant assay in an attempt to identify escape mutants. The assay classified 1.6% of specimens as mutants and another 1.4% as possible mutants, of which 65.5% and 29.2% were confirmed by DNA sequencing respectively. However, the prevalence of escape mutants was underestimated by the assay because it was not designed to identify escape mutations outside of E1 (aa 120–124) and E2 (aa 139–147). DNA sequencing of specimens classified as wild type by the mutant assay revealed 13% harbored escape mutations, of which 64% fell outside of E1 and E2. In addition, many infections that were a mixture of mutant and wild type had a wild type phenotype in the mutant assay. While the mutant assay used in this study enhanced identification of epitope 1 and 2 mutants, effective serological detection of HBsAg mutants would be improved by the use of a broader range of MAbs with well-defined epitopes. The alternative of using HBV DNA sequencing to identify mutants also has its limitations. When the DNA sequence indicates a mixture of wild type and mutant viruses, there is no way to know the relative expression level of HBsAg from each virus. In this study, 39% of mutations were mixtures of wild type and mutant amino acids and fall into this category. In addition, HBsAg antigenicity cannot always be inferred from the DNA sequence as genetic background at distant amino acid positions can play a role in “a” determinant structure and epitope recognition [24]. We observed that HBsAg epitope profiles did not always correlate with the DNA derived amino acid sequence, as has been noted by others previously [24]. Despite these limitations, the combined use of serological and molecular methods allowed identification of immune escape mutants in Pakistani blood donors. The most common mutations were found in E2 at amino acid positions 143, 144, and 145, which have been shown to impact diagnostic assay sensitivity, as has P120S the most common E1 mutant observed in this study [15, 25]. While newer assay versions show improved detection of mutants, ongoing assessment of the impact of mutants on sensitivity must continue [25, 26]. Notably, all of the samples with immune escape mutations were detected by the ARCHITECT HBsAg Qualitative diagnostic test.

Pakistan initiated a childhood HBV vaccination program in 2002 and has achieved 72% to 87% coverage of children [7, 8, 27]. The vaccination rate for the general adult population is low (1.2%) compared to health care workers (73%) [5, 28]. Since the blood donations in our study were collected from 2009 to 2013 from donors 18 years of age or older, the majority of our population would have been born at least 7 years before childhood HBV vaccination was implemented and are unlikely to be vaccinated. Therefore we conclude that the escape mutants present in the donor population are driven by naturally occurring immunological response rather than by vaccine escape. The 14% prevalence of escape mutants is similar to reports from other countries. A study conducted in four African countries, with largely unvaccinated populations, found a 7.7% prevalence of escape mutants in adults [29]. In a study of HBV chronic infections in Spain, 12.5% of patients harbored strains with one or more mutations associated with escape and an additional 26% had other amino acid substitutions within the “a” determinant [30].

As mentioned previously, reports of the HBV genotypes present in Pakistan vary widely [19]. Our data indicated regional differences but agreed with the overall consensus that genotype D strains predominate. Collectively, all subgenotypes of D accounted for 91% of the HBV strains with 97.3% at AFIT (northern Punjab province) and 82.5% at HBB (southern Sindh province). Subgenotypes of A accounted for 6.3% overall with 1.8% and 12.5% at AFIT and HBB, respectively. Genotype C was previously reported as the most prevalent genotype in the Punjab province [20], but we found it in just 1.5% of all sequences and only at the HBB site. The absence of genotype B in our population may indicate that B is primarily found in the Balochistan province [20]. Due to due to the predominance of genotype D1 in this population, no clear patterns in the relationship between genotype and observed mutation frequencies could be identified.

## Conclusions

The frequency of immune escape HBV mutants, in what we believe is a largely unvaccinated population, highlights the need for more studies into the prevalence of escape mutants. The diversity and impact of HBsAg mutations should be considered in the selection of the assay used for screening. While commercial assays have addressed previously identified mutant detection issues, continued vigilance of their sensitivity must be maintained. Differences between vaccinated and unvaccinated populations, the correlation of escape mutant frequency with genotype, and the impact of escape mutations in different genotype backgrounds on the performance of commercially available HBsAg assays will be important areas for future research.

## Materials and methods

### Study population

Collection of HBsAg reactive blood donor specimens for use in HBV research was approved by the Research Ethics Committee at the Pakistan Medical Research Council (Ref. F4-87/NBC/EC-Project-13/852). Specimens were collected at the Armed Forces Institute of Transfusion (AFIT) blood bank in Rawalpindi and Hussaini Blood Bank (HBB) in Karachi from 2009 to 2013. The blood banks used the ARCHITECT® HBsAg Qualitative (Abbott Diagnostics, Wiesbaden, Germany) assay to screen 706,575 blood donations for the presence of HBsAg. Based on volume availability, 2055 HBsAg specimens were randomly selected for the study.

### HBsAg mutant assay

Specimens were screened for potential HBsAg mutants using an automated research assay based on the Abbott ARCHITECT HBsAg Qualitative assay. The mutant screen assay employs 3 different magnetic microparticles each coated with an anti-HBsAg monoclonal antibody (MAb) to capture antigen present in the donor plasma specimen. Captured antigen is then detected using anti-HBsAg polyclonal antibody conjugated with the chemiluminescent compound acridinium. MAbs to epitope 1 (amino acids 121–124), epitope 2 (139–145) and a third conformational epitope from two different regions of HBsAg (112–117 and 187–207) were used. Specimens were classified as mutant, possible mutant, non-reactive or wild type based on the relative signal to noise ratios for the three MAbs.

### HBV PCR amplification and DNA sequencing

Total nucleic acid was extracted from 200µl plasma using the QIAamp® DNA Blood Mini QIAcube Kit and the QIAcube (Qiagen Inc., Valencia, CA) and eluted in 100µl nuclease free water. First-round and nested PCR amplification were performed using the GeneAmp PCR kit with AmpliTaq DNA polymerase (Applied Biosystems, Carlsbad, CA). First round primers targeting the S gene were either HBV-3194F (ACASTCATCCTCAGGCCATGCAGTGG) or HBV-13F (CCACCAARCTCTGCWAGATCCCAGAG) with reverse primer HBV-1161bR (TTGCCGRGCAACGGGGTAAAGG) amplifying a fragment of 1189 or 1149 base pairs in length, respectively. Second round primers were either HBV-13F or HBV-56F (CCTGCTGGTGGCTCCAGTTC) with reverse primer HBV-1003R (GCGGCAAACCCCAAAAGACC) amplifying a fragment of 1010 or 967 base pairs, respectively. The first round reaction mixture (50 µl) contained 0.4 µM of each primer, 0.25 mM each dNTP, and 20 µl of nucleic acids. First round amplification consisted of denaturation at 94°C for 2 minutes, followed by 10 cycles of 94°C for 30 seconds, 50°C for 30 seconds, and 72°C for 90 seconds and 30 cycles of 94°C for 30 seconds, 60°C for 30 seconds, and 72°C for 90 seconds with a final extension at 72°C for 10 minutes. The nested PCR reaction (50 µl) contained 0.4 µM of each primer, 0.25 mM each dNTP, and 2 µl of the first round PCR reaction. Amplification consisted of denaturation at 95°C for 1 minute, 40 cycles of 94°Cfor 15 seconds, 55°C for 30 seconds, and 72°C for 90 seconds, and a final extension at 72°C for 10 minutes.

PCR products were purified using Illustra ExoProStar Purification (GE Healthcare UK Limited, Buckinghamshire, UK) and sequenced directly using the Big Dye Terminator Cycle Sequencing Ready Reaction kit v3.1 and the ABI 3130xl Genetic Analyzer (Applied Biosystems). Sequence data was analyzed using Sequencher 5.2.3 (Gene Codes Corp., Ann Arbor, MI).

### HBV DNA sequence analysis

To determine genotype, HBV DNA sequences were aligned with genotype reference sequences using the CLUSTAL method (MegAlign, Lasergene, DNASTAR Inc., Madison, WI) and manually edited in BioEdit Sequence Alignment Editor (version 7.2.5) [31]. Phylogenetic analysis was performed using PHYLIP (version 3.5c; J. Felsenstein, University of Washington, Seattle, WA). Evolutionary distances were estimated with Dnadist (Kimura two-parameter method) and phylogenetic relationships were determined by Neighbor (neighbor-joining method). Branch reproducibility of trees was evaluated using Seqboot (100 replicates) and Consense. Programs were run with default parameters. Trees were visualized using FigTree (version 1.4.2; A. Rambaut, Institute of Evolutionary Biology, University of Edinburgh, Edinburgh) with chimpanzee sequence AF242585 as an outgroup. Sequences basal to a genotype branch were examined for recombination breakpoints with SimPlot (version 3.5.1; S. Ray,Johns Hopkins University, Baltimore, MD)[32].

The following genotype reference strains (genotype–Genbank accession number) were used in the alignments: A1-AF297623, A1-AF418676, A1-EU366129, A1-M57663.2, A1-U87742.3, A2-AF297624, A2-AP007263, A2-V00866, A3-AB194951, A3-GQ161813, A5-FJ692554, A5-FJ692555, A6-GQ331046, A6-GQ331047, A6-GQ331048, B-D00329, B-D00331, B-D50522, B-M54923, C-D23681, C-D23682, C-X04615, C-X75656, D-X59795, D-Y07587, E-AB194947, E-AB194948, E-AP007262, E-X75657, E-X75664, F-AB036910, F-X69798, F-X75658, F-X75663, G-AB056514, G-AB375165, G-AP007264, G-DQ207798, H-AP007261, H-AY090454, H-AY090457, H-AY090460, I-AB231908, I-FJ023664, A3-AB194952, A5-FJ692556, G-AB064310, G-AF160501, D1-AB104711, D1-AF121239, D1-AF151735, D1-AF280817, D1-AY721611, D1-AY741796, D1-AY945307, D1-X02496, D2-AB078033, D2-AY090453, D2-AY341335, D2-X72702, D3-AJ344117, D3-AY233296, D3-AY373430, D3-DQ111987, D3-X85254, D4-AB033559, D4-AB048702, D5-AB033558, D5-DQ315780, Pan-CHIMP-2-AF242585.

HBsAg subtype was determined as described in Purdy et al. [33]. HBsAg escape mutations were identified using the web-based program Geno2pheno (hbv) v2.0 [23].
